# Early Life Stress Restricts Translational Reactivity in CA3 Neurons Associated With Altered Stress Responses in Adulthood

**DOI:** 10.3389/fnbeh.2019.00157

**Published:** 2019-07-11

**Authors:** Jordan Marrocco, Jason D. Gray, Joshua F. Kogan, Nathan R. Einhorn, Emma M. O’Cinneide, Todd G. Rubin, Thomas S. Carroll, Eric F. Schmidt, Bruce S. McEwen

**Affiliations:** ^1^Harold and Margaret Milliken Hatch Laboratory of Neuroendocrinology, The Rockefeller University, New York, NY, United States; ^2^Department of Neurobiology and Behavior, Stony Brook University, Stony Brook, NY, United States; ^3^Albert Einstein College of Medicine, Bronx, NY, United States; ^4^Bioinformatics Resource Center, The Rockefeller University, New York, NY, United States; ^5^Laboratory of Molecular Biology, The Rockefeller University, New York, NY, United States

**Keywords:** early life stress, CA3 neurons, epigenome- and transcriptome-wide association studies, H3K9me3–Histone H3 tri-methylated at Lysine 9, BAC-TRAP

## Abstract

Early life experiences program brain structure and function and contribute to behavioral endophenotypes in adulthood. Epigenetic control of gene expression by those experiences affect discrete brain regions involved in mood, cognitive function and regulation of hypothalamic-pituitary-adrenal (HPA) axis. In rodents, acute restraint stress increases the expression of the repressive histone H3 lysine 9 tri-methylation (H3K9me3) in hippocampal fields, including the CA3 pyramidal neurons. These CA3 neurons are crucially involved in cognitive function and mood regulation as well as activation of glucocorticoid (CORT) secretion. CA3 neurons also exhibit structural and functional changes after early-life stress (ELS) as well as after chronic stress in adulthood. Using a protocol of chronic ELS induced by limited bedding and nesting material followed by acute-swim stress (AS) in adulthood, we show that mice with a history of ELS display a blunted CORT response to AS, despite exhibiting activation of immediate early genes after stress similar to that found in control mice. We find that ELS induced persistently increased expression of the repressive H3K9me3 histone mark in the CA3 subfield at baseline that was subsequently decreased following AS. In contrast, AS induced a transient increase of this mark in control mice. Using translating ribosome affinity purification (TRAP) method to isolate CA3 translating mRNAs, we found that expression of genes of the epigenetic gene family, GABA/glutamate family, and glucocorticoid receptors binding genes were decreased transiently in control mice by AS and showed a persistent reduction in ELS mice. In most cases, AS in ELS mice did not induce gene expression changes. A stringent filtering of genes affected by AS in control and ELS mice revealed a noteworthy decrease in gene expression change in ELS mice compared to control. Only 18 genes were selectively regulated by AS in ELS mice and encompassed pathways such as circadian rhythm, inflammatory response, opioid receptors, and more genes included in the glucocorticoid receptor binding family. Thus, ELS programs a restricted translational response to stress in stress-sensitive CA3 neurons leading to persistent changes in gene expression, some of which mimic the transient effects of AS in control mice, while leaving in operation the immediate early gene response to AS.

## Introduction

Early life stress (ELS) and stress during adulthood have been associated with an increased prevalence of mood disorders (Felitti et al., 1998; Duman and Monteggia, 2006). Adults that experienced significant abuse or neglect as children have impairments in cognition and memory, change in functional connectivity, decreased hippocampal volumes and impaired reactivity of the hypothalamic-pituitary-adrenal (HPA) axis (King and Laplante, 2005; Tottenham, 2014; Humphreys et al., 2019; Raymond et al., 2018; Barch et al., 2019). Rodent models of ELS have been shown to recapitulate many of these changes, notably impaired learning and memory as well as altered structural and functional plasticity in the hippocampus, a stress-responsive brain region implicated in memory, mood and HPA regulation. Importantly, these changes persist long after the end of the stressor (Maccari et al., 2014). This study seeks to understand the gene expression change that occur in the hippocampus after ELS and how these changes persist to modify brain function in adulthood and reactivity to subsequent stressors.

HPA axis function and release of cortisol (CORT) is modulated by feedback from extra-hypothalamic regions including hippocampus, prefrontal cortex and amygdala that are vulnerable to changes induced by ELS and involve mediators of stress, including the prolonged or maladaptive elevation of glucocorticoid hormones. Neurons in the CA3 region of the hippocampus express high levels of glucocorticoid and mineralocorticoid receptors and exhibit structural and functional changes after ELS, including decreased spine density on CA3 neurons, and accelerated maturation of mossy fiber pathway neurons (Wang et al., 2011; Bath et al., 2016). These changes underlie impaired water maze performance and novel object recognition in ELS-exposed mice (Rice et al., 2008; Bath et al., 2017). Human studies have also shown decreased hippocampal volume and impairments in hippocampus-dependent memory tasks after ELS (Humphreys et al., 2019).

Stress-induced changes in neuronal structure and function have been linked to differences in neuronal gene expression that may involve CORT-induced activation or inhibition of gene expression (Gray et al., 2017). ELS leads to transient and long-lasting gene expression changes in the hippocampus, ventral tegmental area and other areas that are involved in modulating stress susceptibility in adulthood (Rice et al., 2008; Peña et al., 2017). These lasting changes may correspond to differences in epigenetic marks, specifically DNA methylation and histone modifications that also persist into adulthood (Anacker et al., 2014; Turecki and Meaney, 2016; Peña et al., 2017). Understanding how gene expression changes in specific brain regions following ELS is important for identifying how ELS can increase the risk of developing a psychiatric disorder later in life.

In the present study, we demonstrate that a previously established paradigm of ELS leads to long-lasting gene expression changes in stress-sensitive CA3 pyramidal neurons. Further RNA-seq analyses demonstrated that mice with a history of ELS exhibited fewer AS-induced gene expression changes than control mice exposed to the same stress in adulthood. Between control and ELS exposed mice, we found differences in gene expression for DNA methylation and histone methylation, demethylation and acetylation that parallels altered gene expression for diverse pathways such as glucocorticoid receptor binding genes and GABA/glutamate genes. These findings provide new insights into causes and possible treatment targets for cognitive and psychiatric disorders that may result from exposure to adverse events in early life.

## Materials and Methods

### Animals

Gprin3-BacTRAP transgenic male mice were generated in the Heintz Lab as previously described (Heiman et al., 2014) and backcrossed onto the C57/Bl6 background strain. Male mice expressing green fluorescent protein (GFP) were crossed with wild type females, and the GFP+ littermates were used for RNA-sequencing experiment. Wild type mice were used for immunohistochemical staining and corticosterone measurements. Animals 10–12 weeks old were group housed (*n* = 4–5) in standard cages (28.5 × 17 × 13 cm) and were kept on a 12-h light-dark cycle in a temperature-controlled room maintained at 21 ± 2°C with food and water available *ad libitum*. All procedures were performed in accordance with the National Guidelines on the Care and Use of Animals and a protocol approved by The Rockefeller University Animal Care and Use Committee.

### Early-Life Stress protocol

ELS was performed as previously described (Rice et al., 2008). At postnatal day 2, dam and pups were transferred to a clean cage with a wire grid placed in the bottom and given one quarter square of white bedding nestlet. Cages were left undisturbed until postnatal day 12 at which point all animals were transferred to clean cages and left with the mother until weaning at postnatal day 21. Control group was treated with the same conditions except they were given a whole nestlet square and no wire mesh. All groups had unstressed, age-matched controls that were killed concurrently.

### Acute Forced-Swim Stress

Acute forced-swim stress (AS) was performed for 6 min in 2 L of water at 25 ± 2°C in a 4 L beaker. Mice were removed, dried, and allowed to recover for 1 h in the home cage before perfusion or cervical dislocation. Control mice were left undisturbed during the AS procedure prior to perfusion or cervical dislocation. The brain of perfused animals was used for immunohistochemical experiments.

### Corticosterone RIA

Corticosterone levels were measured from plasma collected on the day of sacrifice (*n* = 7–11 mice/group) using a Corticosterone Double Antibody RIA kit (MP Biomedicals Inc., Santa Ana, CA, USA). Briefly, trunk blood was collected in K3 EDTA (K3E) 12 mg Blood Collection Tubes (BD Vacutainer, Franklin Lakes, NJ, USA) 1 h after exposure to AS or from mice left undisturbed during the AS procedure. Samples were then centrifuged at 1,000 *g* for 15 min to collect plasma. Five microliter of plasma [diluted 1:200 in phosphosaline gelatin buffer (pH 7.0 ± 0.1)] and 100 μL of standard calibrators were incubated for 2 h with radioactive corticosterone I^125^ (7 μCi per vial) and then centrifuged at 1,000 *g* for 15 min. Radioactivity in the resulting precipitant was measured using a Hidex Automatic Gamma Counter (Turku, Finland). Corticosterone concentration was calculated using the count per minute (CPM) as a function of the logarithmic equation generated from the calibrators.

### Immunohistochemistry

Seventy to 90 day-old mice (*n* = 5–6 mice/group) were anesthetized with sodium pentobarbital (Nembutal, i.p., 250 mg/kg; Akron Inc., Lake Forest, IL, USA) and transcardially perfused with heparinized saline and 4% PFA. Brains were post-fixed in PFA overnight, transferred to 30% sucrose for 24 h, and stored in O.C.T Compound (Tissue-Tek™). Brain was sectioned at 40 μm on a cryostat (Leica; Buffalo Grove, IL, USA) and stored in cryoprotectant at −20°C prior to labeling. Free floating dorsal hippocampal sections (Bregma: −1.70 to −1.82 mm; 40 μM thick) were processed as follows: washed in 0.01 M phosphate buffered saline (PBS) 3 × 5 min each. Blocked in 0.5% bovine serum albumin (BSA) in 0.01 M PBS, with 0.25% Triton X-100, for 30 min and then incubated in histone H3 lysine 9 tri-methylation (H3K9me3) primary antibody (1:1,000; Millipore, Burlington, MA, USA) in blocking solution at 4°C overnight with shaking. Tissue was rinsed 0.01 M PBS 3 × 10 min each. The sections were then incubated with secondary antibody (Alexa Fluor 488 goat anti-rabbit) diluted 1:1,000 in PBS for 1 h at room temperature and washed in 0.01 M PBS 3 × 5 min each. The sections were incubated with DAPI diluted 1:1,000 in 0.01 M PBS for 5 min and rinsed in 0.01 M PBS 3 × 5 min. The sections were immediately mounted with Prolong Diamond (Thermo Fisher Scientific, Waltham, MA, USA) and cured overnight at room temperature prior to imaging. Investigators were blinded to the groups and sections were photographed at 4× (Supplementary Figure S1) and 20× magnification using a 800 ms exposure on a Nikon Eclipse 90i microscope (Supplementary Figures S1, S2). One section per mouse was quantified at 20× using the Nikon Imaging Suite software tools to calculate mean fluorescent intensity (arbitrary units) for regions of interest dorsal dentate gyrus (DG), CA3, and CA1.

### RNA Sequencing

Seventy to 90 day-old mice were killed by cervical dislocation and rapidly decapitated to extract the hippocampi for translating ribosome affinity purification (TRAP) protocol of CA3 pyramidal neurons. TRAP was performed as described in Gray et al. (2018). A total of 200 ng of RNA per group was prepared for sequencing by The Rockefeller University Genomics Core Facility using the Tru-Seq RNA-Sample Preparation Kit v2 (Illumina, USA). The samples were sequenced on Illumina HiSeq 2500 in a single lane to obtain 100-bp single-end reads at an approximate sequencing depth of 25–35 million reads per sample. The libraries were barcoded to allow for multiplexing within a cell lane. Three biological replicates were used per experimental group, comprising of RNA pooled from 5–6 mice each. At the time of sequencing, experimental groups consisted of three replicates per condition. Each replicate included a unique pool of 5–6 mice, so that each condition comprised a total *n* = 15–18 mice.

### Sequencing Analysis and Statistics

Raw reads were trimmed, filtered, and aligned to mouse genome (10 mm) using Rsubread’s subjunc method (Liao et al., 2013) and exported as bigWigs normalized to reads per million using the rtracklayer package. Transcript expression was calculated using the Salmon software quantification (Patro et al., 2017) and gene expression levels as TPMs and counts were retrieved using Tximport (Love et al., 2016). Differential expression analysis was conducted with DESeq2 to quantify transcript reads and obtain fold change values for individual genes, and the differential transcript and exon usage were evaluated using DEXSeq and Drimseq software (Soneson et al., 2015). Genes with *p* < 0.05, Benjamini–Hochberg false discovery rate corrected, were selected for further analysis. Heatmaps and Principal Component Analysis (PCA) were created using rlog transformed data with batch correction from the limma package (Ritchie et al., 2015; Love et al., 2018). PCA showed that groups were clustered according to their experimental manipulations (Supplementary Figure S3). Heatmaps were generated using the heatmap tool from MeV^1^. Differences in log2 fold change were visualized against the mouse genome by using the Venn diagram function of the BioVenn website^2^. GO categories were manually curated from results of the Database for Annotation, Visualization and Integrated Discovery (DAVID) functional annotation cluster tool. Microsoft Excel (Microsoft, USA) was used to obtain gene expression profiles by sorting genes based on fold change. Corticosterone data and immunohistochemistry data were analyzed using GraphPad Prism (GraphPad Software Inc., San Diego, CA, USA) by performing a two-way ANOVA followed by Neumann–Keuls *post hoc* analysis. A *p*-value < 0.05 was set as statistically significant. RNA-sequencing data are deposited in GEO (GSE131972). All other relevant data are available from the authors upon reasonable request.

## Results

### ELS Alters Neuroendocrine Response to Acute Stress in Adulthood

**Figure 1:**
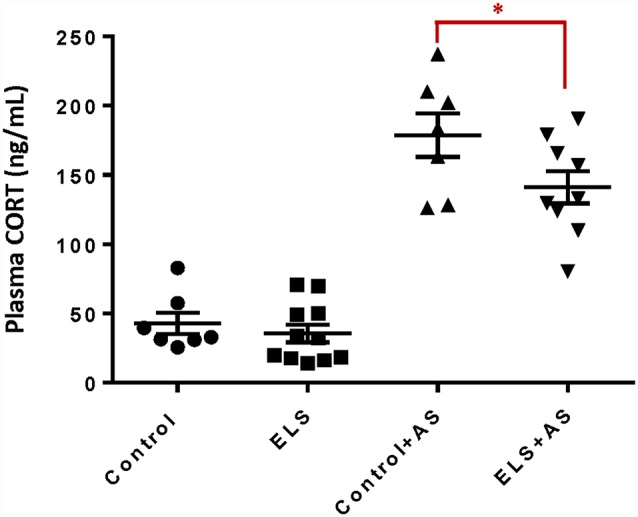
Reduction of corticosterone levels in ELS mice induced by acute stress. Bar chart shows that unstressed controls and ELS mice have comparable levels of plasma corticosterone. ELS mice show a significant decrease after acute stress when compared to controls after acute stress. Mice exposed to AS regardless of perinatal manipulation exhibit higher levels of CORT relative to unstressed mice. *p < 0.05, ELS, early life stress (vertical lines). AS, acute stress (diagonal lines; Control: *n* = 7, AS: *n* = 7, ELS: *n* = 11, ELS + AS: *n* = 9 mice).

ELS is associated with alterations in stress responsiveness in both humans and animal models (Rice et al., 2008; Weinstock, 2008; Maccari et al., 2014; Raymond et al., 2018). To assess the influence of ELS upon the stress response during adulthood, we measured the plasma levels of CORT after acute forced swim stress (AS), a well-validated paradigm that rapidly increases CORT levels following a 6-min swim challenge (Bohacek et al., 2015; Molendijk and de Kloet, 2019). ELS alone did not change basal levels of CORT in adult mice. One hour after AS, both controls and ELS mice displayed increased CORT levels. However, ELS mice exhibited lower, blunted CORT levels when compared to controls after AS (*F*_(1,30)_ = 4.46, *p* < 0.05; Figure 1) which may suggest an impaired ability to regulate HPA function (Dunn and Orr, 1984).

### Levels of H3K9me3 in Hippocampus After ELS

Stressful experiences at different stages of development, in both rodents and humans, have been associated with changes in gene expression under the control of epigenetic regulation (Weaver et al., 2004a,b; Korosi and Baram, 2009; Watamura and Roth, 2018). For example, acute stress increases the expression of the repressive histone marker H3K9 tri-methylation (H3K9me3) in the CA1 and DG regions of the rat dorsal hippocampus (Hunter et al., 2009). To assess the compound effects of ELS and acute stress in adulthood on methylation, we investigated the levels of H3K9me3 in the hippocampus of control and ELS mice exposed to a paradigm of acute swim stress (AS). Immunofluorescence of CA1, CA3, and DG neurons, showed that the effects of ELS were region specific in the dorsal hippocampus. In CA3 neurons, ELS increased the levels of H3K9me3 when compared to unstressed control mice (ELS-by-AS: *F*_(1,18)_ = 26.92, *p* < 0.01). Furthermore, in ELS mice, AS induced a significant downregulation of H3K9me3 when compared to unstressed ELS mice (*p* < 0.01), whereas AS induced an upregulation of H3K9me3 in control mice (*p* < 0.01). Of note, AS in ELS mice produced a level of H3K9me3 that was lower than that in control mice after AS (*p* < 0.05).

**Figure 2:**
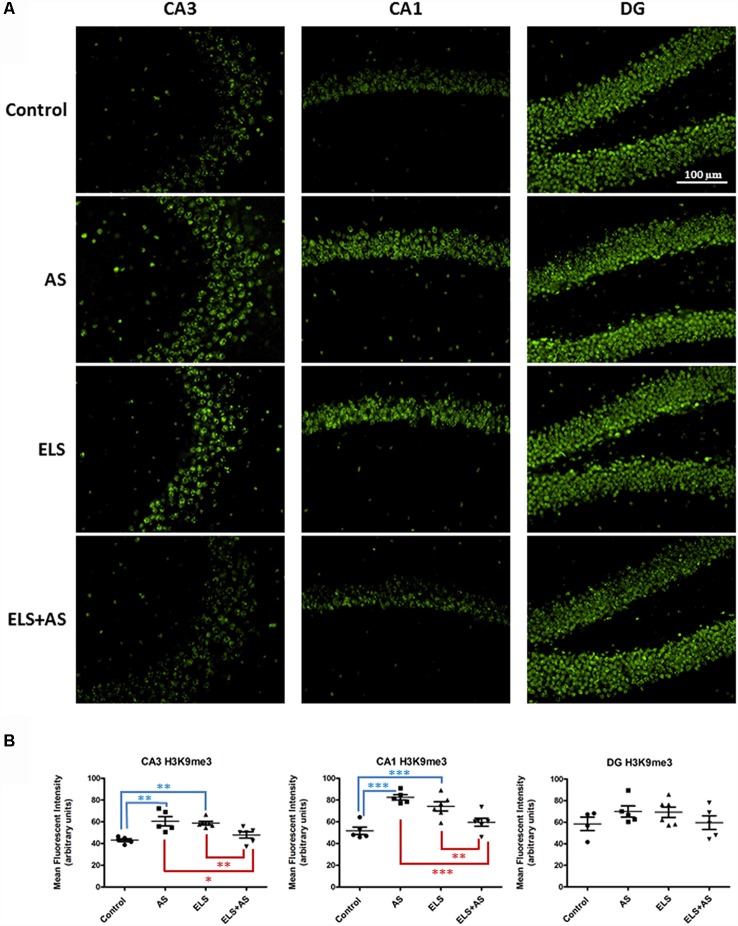
Effects of AS and ELS on histone H3 lysine 9 tri-methylation (H3K9me3) levels in the hippocampus. **(A)** Immunofluorescence at 20× magnification was used to detect H3K9me3 in CA1, CA3, and dentate gyrus (DG) regions of the dorsal hippocampus. Compared to unstressed controls, ELS and stressed controls exhibit an increase in H3K9me3 in both CA3 and CA1 regions. ELS mice subjected to AS show a reduction in H3K9me3 expression when compared to unstressed ELS mice and stressed controls in CA1 and CA3. AS does not induce differential H3K9me3 expression in control and ELS mice in the DG. Scale bar = 100 μm. **(B)** Scatter plots show quantification of H3K9me3 immunoreactivity (*n* = 5–6). *p < 0.05, *p < 0.01, ***p < 0.001. ELS, early life stress; AS, acute stress. Control: *n* = 5, AS: *n* = 5, ELS: *n* = 6, ELS + AS: *n* = 6 mice.

Similarly to CA3, CA1 regions of control mice exhibited increased H3K9me3 levels after AS relative to unstressed controls and also compared to unstressed ELS mice (ELS-by-AS: *F*_(1,18)_ = 39.84, *p* < 0.001). ELS also increased H3K9me3 levels in CA1 neurons, whereas AS in ELS mice decreased expression of H3K9me3 (*p* < 0.01) compared to both ELS mice and stressed controls (*p* < 0.001). Thus, AS had an opposite effect on H3K9me3 in controls and ELS mice in both CA1 and CA3 neurons. We observed no differences in the dorsal DG, suggesting that the effects of acute stress and ELS were specific to CA1 and CA3 regions (Figures 2A,B).

The expression of the activating mark H3K4me3 was also measured in CA1, CA3, and DG of unstressed controls and ELS mice. No differences were found in the levels of H3K4me3 (Supplementary Figures S4A,B). We then investigated, by whole genome sequencing analysis, whether repression of gene expression is a dominant result of ELS, focusing upon the CA3 neurons because of their sensitivity and vulnerability to stress.

### Epigenetic Signature in CA3 Neurons in Response to ELS

ELS causes reduced spine density in CA3 neurons, but not in CA1 neurons (Wang et al., 2011). Moreover, CA3 neurons are involved in key memory functions of the hippocampus (Lisman et al., 2005) as well as stimulation of HPA activity (Dunn and Orr, 1984). Previous work showed that the translational profile of mice subjected to different types of stress displays distinct gene pathways within CA3 pyramidal neurons (Marrocco et al., 2017; Gray et al., 2018). Thus, in order to assess the effects of AS and ELS on the translational profile of CA3 pyramidal neurons, we used the TRAP method to isolate cell type-specific translating mRNAs. BAC transgenic mice expressing EGFP-tagged ribosome protein L10a (EGFPL10a) specifically in CA3 pyramidal cells were used. EGFP-tagged polysomes were immunoprecipitated (IP) from hippocampal homogenates and bound mRNA was isolated and analyzed by high throughput RNA sequencing (Heiman et al., 2014). While RNA-seq data does not allow for the interrogation of histone and DNA modifications, alterations of common enzymes involved in creating and perpetuating epigenetic modifications were observed in CA3 pyramidal neurons of control and ELS mice exposed to AS.

**Figure 3:**
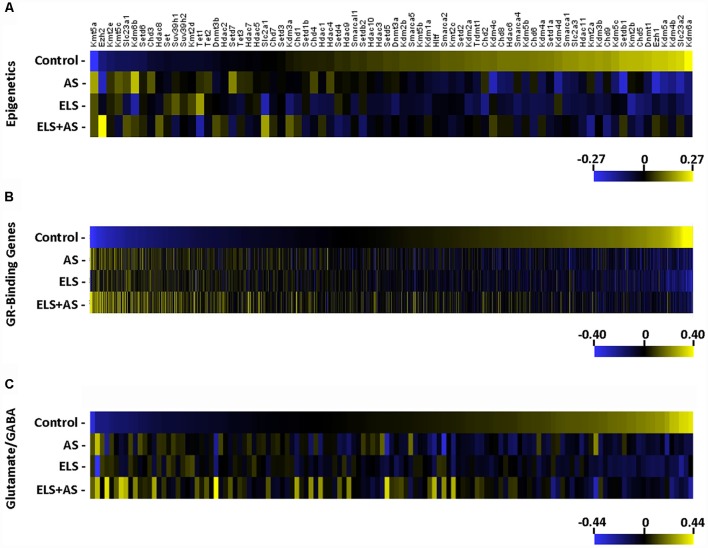
Regulation of gene expression induced by AS and ELS. **(A–C)** Heatmaps represent the log2 transformation of counts from 74 epigenetic genes, 1,506 GR-binding genes, and 127 glutamate/GABA genes. Genes are organized by lowest to highest expression in the unstressed control group. The majority of genes in each heatmap is downregulated in stressed controls and unstressed ELS mice, but unchanged in stressed ELS mice. ELS, early life stress; AS, acute stress.

Seventy-four epigenetic genes including ATP-dependent chromatin remodelers, chromatin helicase DNA binding proteins, DNA methylation and demethylation, histone deacetylases, histone demethylases, and histone methyltransferases genes (Zhu et al., 2016) were clustered into a heatmap. A majority of epigenetic genes were downregulated by AS in controls and in unstressed ELS mice when compared to unstressed controls. Notably, many of the same epigenetic genes that were altered by AS in controls were unresponsive to AS in ELS mice (Figure 3A).

The hippocampus is known to be particularly sensitive to ELS-glucocorticoid epigenetic regulation (Weaver et al., 2004a,b) and stress-induced glucocorticoid-binding genes in CA3 neurons (Marrocco et al., 2017), resulting in mice neuronal functional modulation. Recently, ChIP-sequencing identified a repertoire of GR-binding genes (GRBG) within the hippocampus that respond to CORT (Polman et al., 2013). These 1,506 GRBGs exhibit two patterns in response to AS and in relation to ELS. A majority of GRBGs were downregulated by AS in controls and in unstressed ELS mice when compared to unstressed controls; yet, many of these GRBGs were unresponsive to AS in ELS mice when compared to unstressed ELS mice.

Another pattern consisted of GRBGs that were upregulated by AS in both controls and ELS mice (Figure 3B). Since pyramidal neurons of the CA3 region are primarily glutamatergic (Spruston et al., 1995) and stress is known to regulate the expression of glutamate-related genes in the hippocampus (Bartanusz et al., 1995), we generated a heatmap of 127 glutamate- and GABA-related genes (Gray et al., 2018). Again, a majority of the glutamate/GABA genes were downregulated by AS in controls and also in unstressed ELS mice when compared to unstressed controls. Again, many of these glutamate/GABA genes were unresponsive to AS in ELS mice (Figure 3C). Thus, epigenetic, GRBGs, and glutamate/GABA clusters all showed similar patterns of response to ELS and to AS.

**Figure 4:**
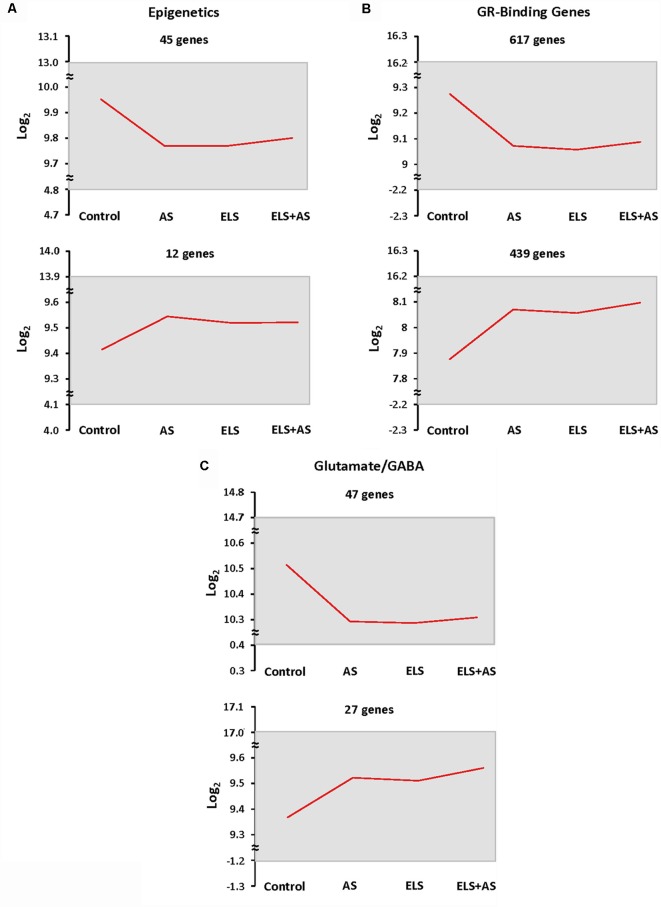
Epigenetic profiling of ELS mice shows a stressed state in the absence of AS. **(A–C)** Clustering of epigenetic, GR-binding, and glutamate/GABA genes by expression profiles shows that many genes are downregulated in every experimental group when compared to unstressed control mice. Genes were clustered based on the log2 transformation of counts in control mice subjected to AS, ELS mice, and ELS mice subjected to AS vs. unstressed control mice. The mean of the log2 transformation of counts is presented as a red line graph with the maximum and minimum values of the cluster represented as a gray rectangle. The top two most populated expression patterns are shown. ELS: mice with a history of early life stress. AS: control mice subjected to acute stress. ELS + AS: ELS mice subjected to acute stress.

In order to count the number of genes that fell in this pattern, we thresholded genes based on the direction of their log2 transformation of counts in stressed controls, unstressed ELS mice, and ELS mice exposed to AS vs. unstressed controls. Genes with the same expression profile were clustered together. When compared to unstressed controls, the number of genes that were downregulated by AS or by ELS alone, which programmed a blunted response to AS, in the epigenetic, GRBGs, and glutamate/GABA clusters was 45, 617, and 47 genes, respectively. Similarly, the number of genes that were upregulated relative to control by AS or by ELS alone in the epigenetic, GRBGs, and glutamate/GABA clusters was 12, 439, and 27 genes, respectively (Figures 4A–C). Again, the downregulated pattern represented the predominant direction of the ELS effect. However, AS or ELS alone resulted in a minority of upregulated genes that will also be discussed below.

### ELS Reduces Translational Response to Acute Stress in CA3 Pyramidal Neurons

In order to assess the translational impact of ELS and AS on the whole genome, we increased the criteria of statistical significance (*p*-value < 0.05, FDR <0.05) when analyzing the RNA-sequencing data of control and ELS mice naïve or exposed to stress. We first evaluated the translational profiles across conditions, i.e., AS in controls, ELS mice, and ELS mice exposed to AS, against naïve controls. This comparison resulted in a consistent number of genes shared by controls after AS and naive ELS mice compared to controls (*n* = 540 genes; Supplementary Figure S5A). Furthermore, filtering these common genes by fold change directionality demonstrated that a majority of differentially regulated genes across conditions were downregulated with respect to unstressed controls (Supplementary Figure S5B).

A GO analysis of downregulated genes included pathways related to synaptic plasticity, metabolism, and glutamate/GABA signaling (Supplementary Figure S5C). Interestingly, within this list, we identified the lysine-specific demethylase 6a, *Kdm6a*, which is epigenetically repressed by methylation (Laukka et al., 2018), consistent with our findings (Figure 3A).

**Figure 5:**
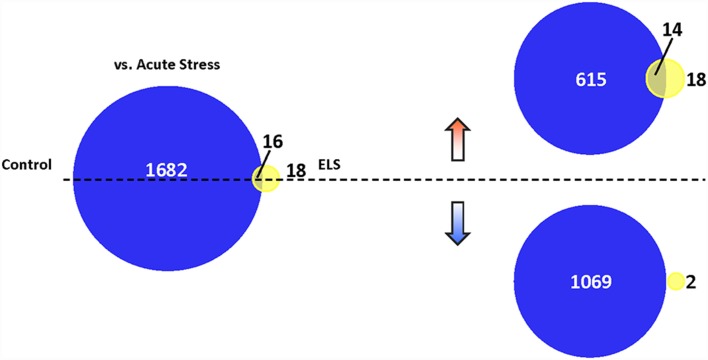
Translational repression in ELS mice following AS. Venn diagram depicting the number of genes altered in stressed control mice vs. unstressed control mice (dark blue), stressed ELS mice vs. unstressed ELS mice (yellow), and in both comparisons (yellow-blue overlap; *p* < 0.05). AS induces a markedly limited gene expression change in ELS mice relative to control mice. We found that AS induces 1,698 genes in control mice and 34 genes in ELS mice, with 16 genes common to both comparisons. Separating these AS-regulated genes based on the direction of their fold change revealed that AS upregulates 629 genes in control mice and 32 genes in ELS mice, with 14 genes commonly upregulated in both comparisons. In addition, 1,069 genes are downregulated in control mice and two genes downregulated in ELS mice by AS, with no genes common to both comparisons. ELS, early life stress; AS, acute stress.

We then assessed the effects of AS in controls and ELS mice, respectively, at a new level of statistical stringency. When filtering genes by fold change directionality, a majority of genes in ELS were upregulated by AS, whereas most genes were downregulated by AS in controls. AS induced 1,698 genes in controls (GO analysis shown in Supplementary Figure S6) and only 34 genes in ELS mice, with 16 genes common to both conditions (Figure 5).

Of note, those common genes that were mostly upregulated by AS in both controls and ELS mice included the immediate early genes such as the Fos-family genes, Egr-family genes, and Arc (Supplementary Table S1). This demonstrated that both controls and ELS mice responded to AS by activating the immediate early gene response, although ELS dramatically blunted the subsequent translational response to stress.

In comparison, the majority of genes that were upregulated by AS in ELS mice were included in the GRBG heatmap prediction, such as *Per1, Dusp1, Nfkbia, Cst3, Trib1, Htra1, Sdc4, Plekhf1* (Figure 2; Supplementary Table S1). This indicates that AS induced translational repression in controls, whereas it led to translational activation of those genes in mice with a history of ELS.

## Discussion

Using a mouse model, we determined the lasting consequences of ELS on stress reactivity and gene expression in hippocampal CA3 pyramidal neurons that are structurally and functionally vulnerable to the effects of stressors and are critically involved in spatial and episodic memory, as well as mood (McEwen, 2001, 2016). We found that ELS profoundly alters gene expression patterns and responsiveness to acute stressors, with the main effect being considerable repression of the ability of acute novel stress to alter gene expression along with a qualitative change in genes that are regulated by an acute novel stressor. The effects of ELS are also manifested in blunted HPA activation and altered patterns of repressive and activating histone methylation marks in hippocampal subfields, with considerable changes and restriction in cell-type specific translational profiling of stress-sensitive CA3 pyramidal neurons.

We demonstrated that ELS exposure leads to a blunted corticosterone activation response after AS in adulthood, pointing to a reprogramming of the HPA axis while brain circuitry is still forming (Lupien et al., 2009). Hippocampal circuitry exerts both positive and negative feedback and inhibitory control over the HPA axis (Joëls et al., 2004) whereby CA3 activation increases HPA axis activity (Dunn and Orr, 1984). The ability to mount an adequate corticosterone response after a stressor is necessary for an organism to coordinate metabolic, immune and other changes that allow for adaptation to a changing environment (McEwen, 2019).

The effects of corticosterone include participation in remodeling of CA3 neuronal dendrites and spine synapses (McEwen, 2016). This utilizes genomic and non-genomic mediation through glucocorticoid receptors [both mineralocorticoid (MR) and glucocorticoid (GR)], which are highly expressed in hippocampal CA3 pyramidal neurons (McEwen, 2016). ELS also affects CA3 neuron structure: using the same paradigm of ELS, Wang et al. (2011) demonstrated decreased spine density in CA3 hippocampal neurons, associated with cognitive deficits and disrupted long-term potentiation selective to those neurons. Neuroanatomical changes of the hippocampus have also been observed in children that experienced adverse events during critical periods of development (Humphreys et al., 2019). Thus, alterations of neuronal architecture combined with attenuation of appropriate neuroendocrine and transcriptional responses to acute stressors may lead to behavioral dysfunctions and cognitive inflexibilities in subjects exposed to adverse events early in life (Walker et al., 2017).

Concerning the effects of acute novel stress (AS) on epigenetic modifications in control and ELS mice, we demonstrated that AS induced an opposite regulation of the repressive histone mark, H3K9me3, in the dorsal CA1 and CA3 regions of control and ELS mice. We note that in naïve rats, as well as mice in our study, acute stress increases H3K9me3 levels (Hunter et al., 2009, 2012). Surprisingly, ELS mice showed chronically elevated levels of H3K9me3 at baseline, which were then decreased following AS; whereas, AS elevated H3K9me3 levels in both dorsal hippocampal regions of control mice.

Modifications to the repressive histone marker at the protein level were paralleled by changes in gene expression in these neurons. *Suv39h1*, a specific H3K9 methyltransferase (Peters et al., 2001), was increased by AS in control and was also elevated in unstressed ELS mice compared to unstressed controls. Remarkably, in ELS mice, AS induced decreased levels of *Suv39h1* compared to ELS controls. Thus, AS and ELS effects are both manifested in the levels of H3K9me3, as well as a key methylating enzyme, in the dorsal hippocampus in a double-hit model of stress. Notably, many other genes included in our bioinformatics pathway analysis showed reduced (or in some cases, elevated) expression in both ELS mice and in control mice as a result of AS. Thus, ELS induces long-lasting programming of CA3 neurons to chronically activate or repress the same translational responses that are acutely activated or repressed by AS in control mice.

Previous work from this laboratory and others have demonstrated that TRAP, the method used in this investigation, has advantages compared to whole tissue mRNA isolation (Marrocco et al., 2017; Gray et al., 2018). We demonstrated strong effects of ELS and AS upon three crucial gene networks, namely: (i) epigenetic modifiers; (ii) GRBGs; and (iii) glutamate/GABA genes. Many genes in these pathways were down-regulated by AS in control mice, and were also decreased by ELS in the absence of any AS when compared to controls; some genes were up-regulated by AS in controls and elevated in unstressed ELS mice compared to controls. Many of those up or down-regulated genes in ELS mice did not respond to AS. This suggests that a history of ELS not only induced considerable persistent translational repression or activation but also led to reduced reactivity to AS.

Genes activated by AS in controls, but not in ELS mice, were found to participate in glutamate/GABA signaling, proteolysis, RNA splicing, and neurodegenerative disease. The glutamate/GABA system is essential for the stress response and regulation of mental state (Popoli et al., 2011). Stress-dependent downregulation of glutamate/GABA genes in control mice is consistent with previous findings showing that acute stress reduces the expression of *Grin1*, *Grin2a*, *Gabbr2*, and *Gabra1* in CA3 neurons (Marrocco et al., 2017). However, ELS mice exhibited no changes in those same glutamate/GABA genes when exposed to AS. The mineralocorticoid receptor, *Nr3c2*, was also reduced by AS in control mice compared to unstressed mice, as also previously reported (Marrocco et al., 2017), but AS did not affect *Nr3c2* expression in ELS mice.

In contrast, the considerably restricted list of genes selectively induced by AS in ELS mice involved cellular functions such as circadian rhythm (*Per1*, *Npy*), inflammatory response (*Nfkbia*), opioid receptors *(Penk)*, and in GRBG function (*Dusp1, Cst3, Trib1, Htra1, Sdc4, Plekhf1*). Glucocorticoid receptors play a key role in the programming of the ELS synapse as well as impaired response to AS (Weaver et al., 2004a,b; Meaney and Szyf, 2005; Mifsud and Reul, 2018). Moreover, transcriptional disruption of circadian rhythms is observed across diverse animal models of early life adversities (Baranger et al., 2016; Nätt et al., 2017; Yam et al., 2017; Morley-Fletcher et al., 2019). *Neuropeptide Y (Npy)* is implicated in the regulation of hippocampal synaptic plasticity and circuit structure in a model of predator scent stress (Li et al., 2017), while *Nfkbia* is involved in stress sensitization and recovery in a double-hit model of stress (Gray et al., 2014). Further research is needed to elucidate the mechanistic consequences of the epigenetic repression of these subsets of genes and changes in circuit connectivity of CA3 neurons in stressed mice with a history of ELS.

Yet it is particularly noteworthy that the reduced translational reactivity to AS of ELS mice did not include the immediate early genes *Egr1/2/4*, *Arc*, *Fos*, and *Fosb*, which were upregulated by AS in both control and ELS mice. Immediate early genes are regarded as the “gateway to the genomic response,” the qualitative nature of which is dependent on other epigenetic factors that determine which genes will respond (Pérez-Cadahía et al., 2011). Interestingly, the activation of immediate early genes, such as *Fos* and Arc, by acute stress occurs in both males and females as well (Marrocco et al., 2017).

In this regard, it is crucial to consider that this study only includes male mice, while it is established that females respond differently to AS or ELS (Bohacek et al., 2015; Bath et al., 2017; Marrocco et al., 2017; Manzano Nieves et al., 2019). Thus, further studies are needed to investigate the genomics of ELS in females that take into account the role of sexual differentiation. Additionally, the TRAP sequencing of hippocampal CA3 neurons is likely to contain cells from both dorsal and ventral hippocampus. It would be of interest then to examine epigenetic markers in the ventral hippocampus because of its distinct neuroanatomical connectivity (Fanselow and Dong, 2010).

Together, our data show that chronic early stress induced by limited bedding and nesting material programs a restricted translational response to stress in stress-sensitive CA3 neurons leading to persistent changes in gene expression. Some of these changes mimic the transient effects of AS in control mice while leaving in operation the immediate early gene response to AS. Alterations in epigenetic modifiers observed in CA3 neurons should be extended to other stress-sensitive neuronal populations to investigate novel brain targets of ELS programming of adult stress reactivity. Such studies pave the way for investigating interventions that may treat and reverse the effects of adverse early life programming.

## Data Availability

The datasets generated for this study can be found in GEO, GSE131972.

## Ethics Statement

This study was carried out in accordance with the recommendations of the National Guidelines on the Care and Use of Animals. The protocol was approved by The Rockefeller University Animal Care and Use Committee.

## Author Contributions

JG, JM, and BM designed the experiments. JG, JM, NE, EO’C, and BM wrote the manuscript. JG, JM, JK, NE, EO’C, and TR performed the experiments. TC contributed to bioinformatics analysis. JM, NE, and EO’C prepared the final figures and performed statistical analysis. ES contributed the BAC-TRAP method.

## Conflict of Interest Statement

The authors declare that the research was conducted in the absence of any commercial or financial relationships that could be construed as a potential conflict of interest.

## Footnotes

^1^http://mev.tm4.org/

^2^http://www.biovenn.nl/index.php

## Supplementary Material

FIGURE S1 Principal Component Analysis (PCA) of Gprin3-Bac TRAP RNA-sequencing data. Unstressed control mice (black), ELS mice (red), control mice subjected to AS (blue), and ELS mice subjected to AS (green) show clustering based on experimental groups. ELS, early life stress; AS, acute stress.

FIGURE S2 H3K9me3 and DAPI levels of control and ELS mice. 4× magnification imaging of immunofluorescence was used to detect H3K9me3 and DAPI in CA1, CA3, and DG regions of the dorsal hippocampus. Column labeled “Merge” depicts both the H3K9me3 and DAPI image overlaid. Scale bar = 100 μm. ELS, early life stress; AS, acute stress. Control: *n* = 5, AS: *n* = 5, ELS: *n* = 6, ELS + AS: *n* = 6 mice.

FIGURE S3 DAPI levels of control and ELS mice. 20× magnification imaging of immunofluorescence was used to detect DAPI in CA1, CA3, and DG regions of the dorsal hippocampus. Scale bar = 100 μm. ELS, early life stress; AS, acute stress. Control: *n* = 5, AS: *n* = 5, ELS: *n* = 6, ELS + AS: *n* = 6 mice.

FIGURE S4 H3K4me3 levels do not change following ELS. **(A)** Immunofluorescence was used to detect H3K4me3 in CA1, CA3, and DG regions of the hippocampus from unstressed controls and ELS mice. Compared to unstressed controls, ELS does not induce differential H3K4me3 expression in the CA1, CA3, and DG. Scale bar = 100 μm. **(B)** Scatter plots show quantification of H3K4me3 immunoreactivity (*n* = 5–6). Control: Unstressed control mice. ELS: Mice with a history of early life stress.

FIGURE S5 ELS mice exhibit loss of translational activation following AS. **(A)** Triple Venn diagram depicting the number of genes altered in stressed control mice (blue), ELS mice (pink), and stressed ELS mice (yellow) when compared to unstressed control mice. **(B)** Genes of the triple venn diagram comparisons separated based on the direction of their fold change. (left) AS (blue) upregulates 629 genes in control mice and ELS mice exhibit (pink) an upregulation of 400 genes, with 318 genes commonly upregulated in both comparisons. In addition, 1,069 genes are downregulated in control mice by AS and 748 genes downregulated in ELS mice, with 603 genes common to both comparisons. (middle) ELS mice exhibit an upregulation of 400 genes and AS induces an upregulation of 301 genes in ELS mice (yellow), with 174 genes common to both comparisons. Furthermore, 748 genes are downregulated in ELS mice and 469 genes downregulated in ELS mice by AS, with 437 genes common to both. (right) AS upregulates 629 genes in control mice and 301 genes in ELS mice, with 184 genes commonly upregulated in both comparisons. AS downregulates 1,069 genes in control mice and 469 genes in ELS mice, with 404 genes common to both comparisons. **(C)** GO analysis of all the genes downregulated in Supplementary Figure S3B. Bar graph chart depicting the enrichment analysis of the top 10 pathways from Database for Annotation, Visualization, and Integrated Discovery (DAVID) revealed gene pathways related to protein transport, phosphorylation, glutamate/GABA, and axon guidance.

FIGURE S6 AS alters cell signaling related to the glutamate/GABA system in control mice. Bar graph chart depicting the enrichment analysis of the top 10 pathways from Database for Annotation, Visualization, and Integrated Discovery (DAVID) revealed gene pathways related to Glutamate/GABA, neurodegenerative disease, and transport, such as protein transport and ion transport. Pathways were obtained by analyzing the genes unique to control mice subjected to AS when compared to unstressed controls (Figure 2B; dark blue circle).

TABLE S1 Genes induced by AS in ELS mice. Table depicts the fold change and *p*-value of 34 genes differentially expressed by AS in ELS mice when compared to unstressed ELS mice. Genes highlighted in blue are genes also present in control mice subjected to AS when compared to unstressed control mice. Genes with a star are immediate early genes (*Egr1*, *Egr2*, *Egr4, Fos*, *Fosb*, and *Arc*).
