# Supplementary material for: Early Life Stress Restricts Translational Reactivity in CA3 Neurons Associated With Altered Stress Responses in Adulthood
Source: Front Behav Neurosci. 2019 Jul 11;13:157. doi: 10.3389/fnbeh.2019.00157 (PMC6637287; doi:10.3389/fnbeh.2019.00157)
Supplement: FIGURE S1 — Principal Component Analysis (PCA) of Gprin3-Bac TRAP RNA-sequencing data. Unstressed control mice (black), ELS mice (red), control mice subjected to AS (blue), and ELS mice subjected to AS (green) show clustering based on experimental groups. ELS, early life stress; AS, acute stress. [file Data_Sheet_1.PDF]

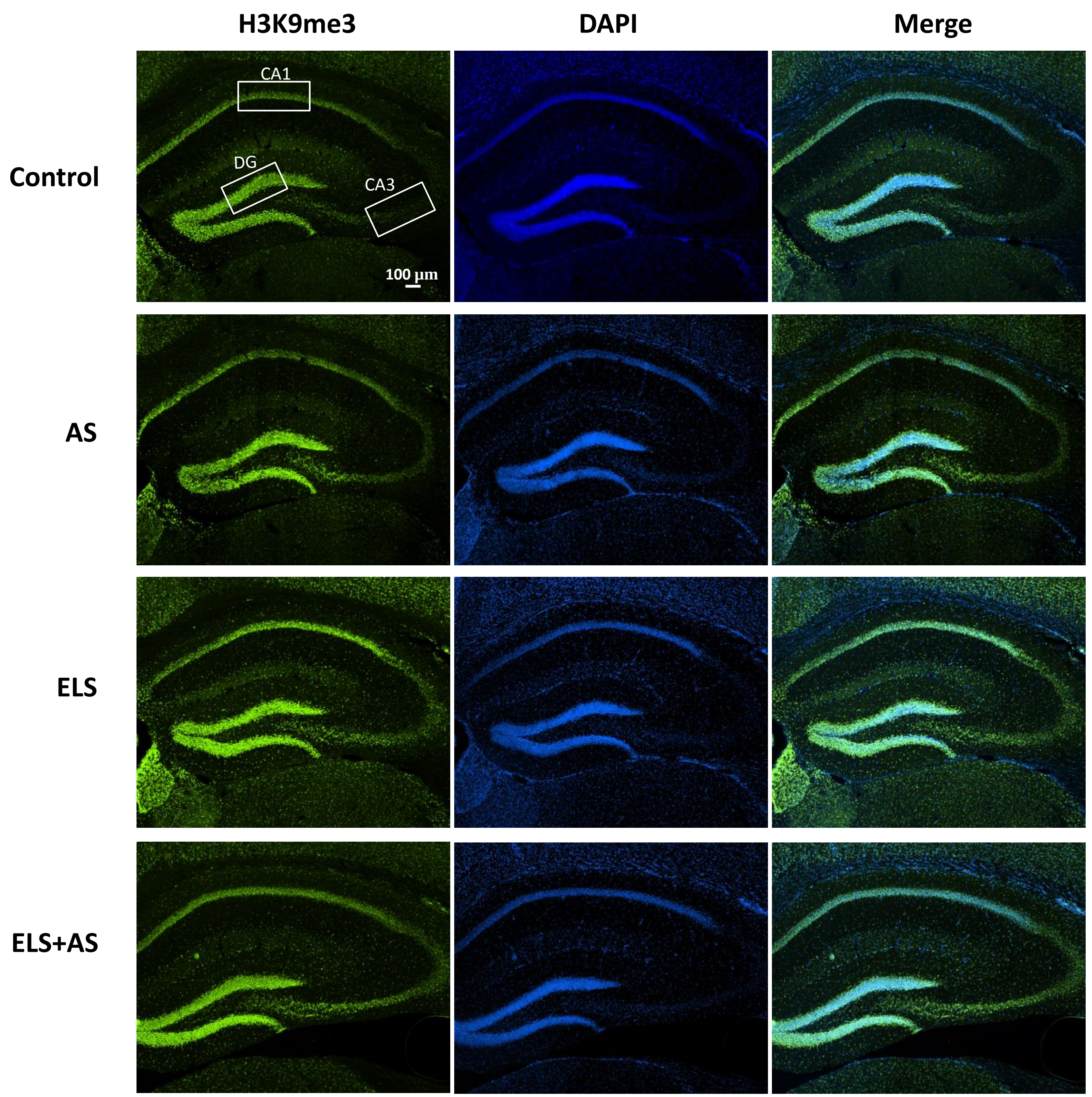

Supplementary Fig. 1

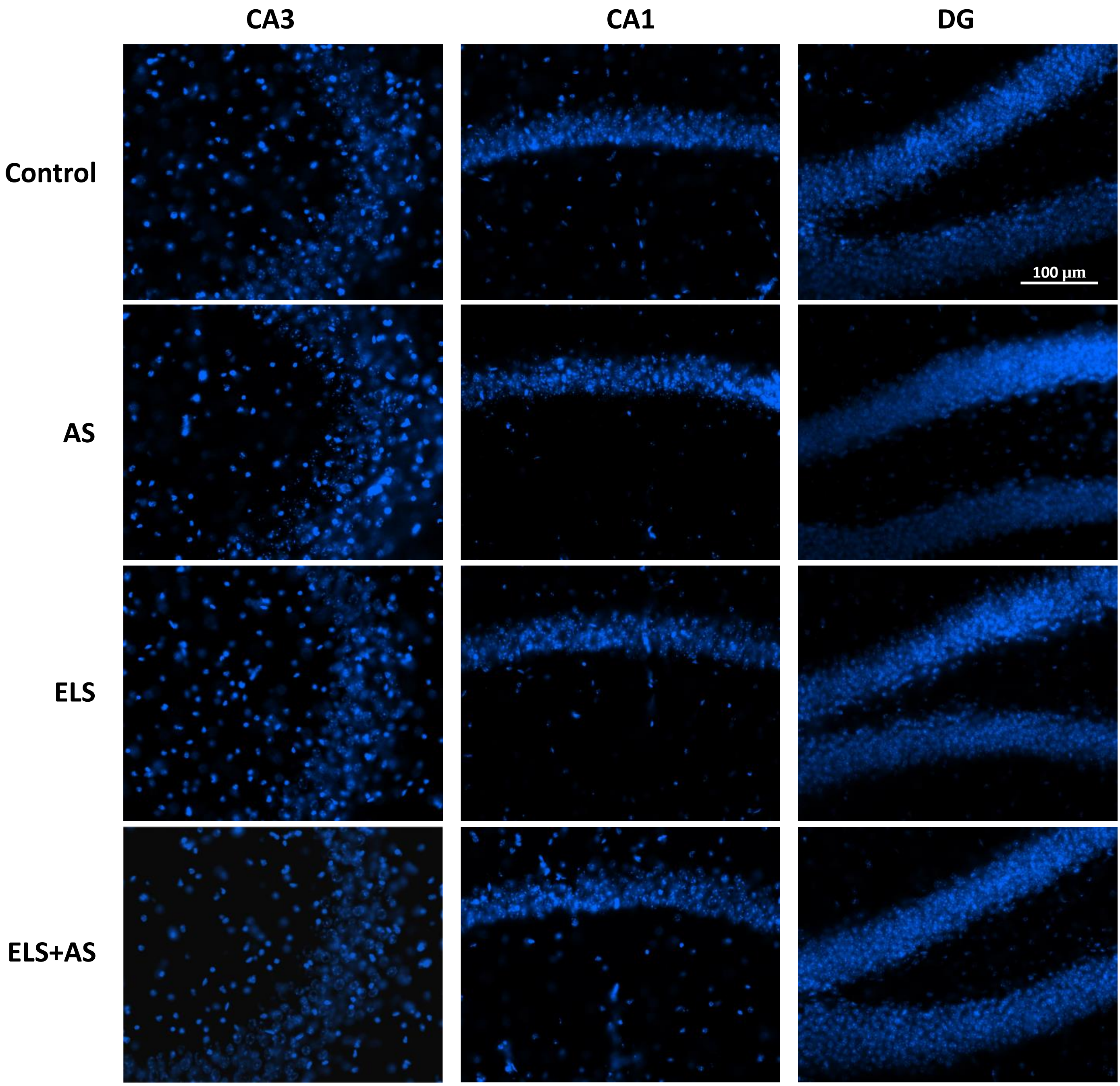

Supplementary Fig. 2

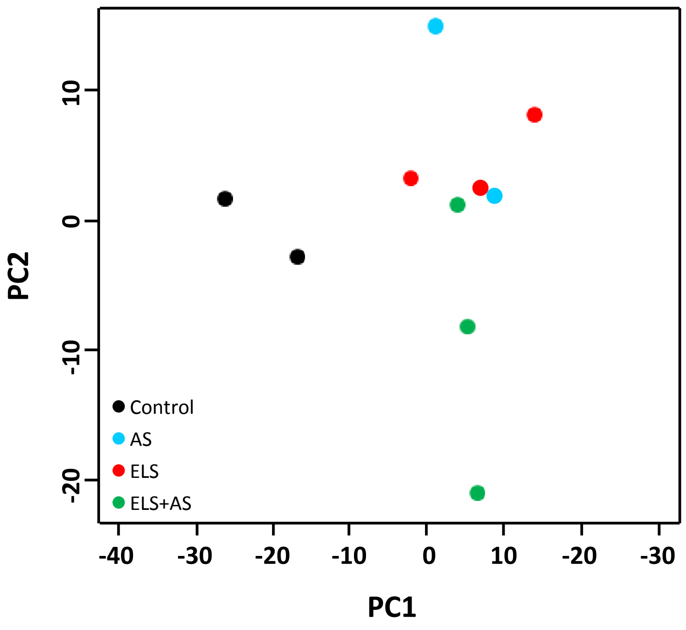

Supplementary Fig. 3

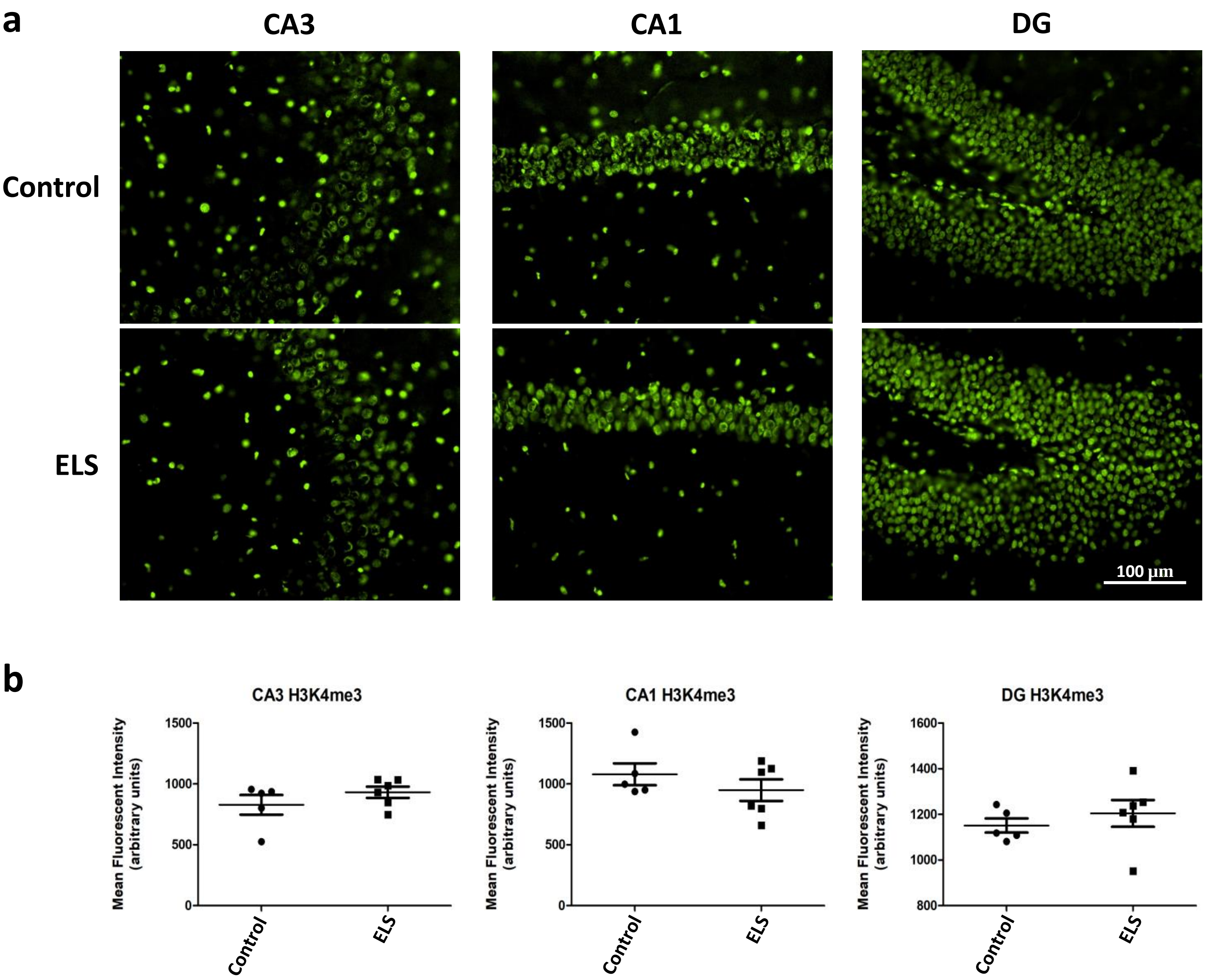

Supplementary Fig. 4

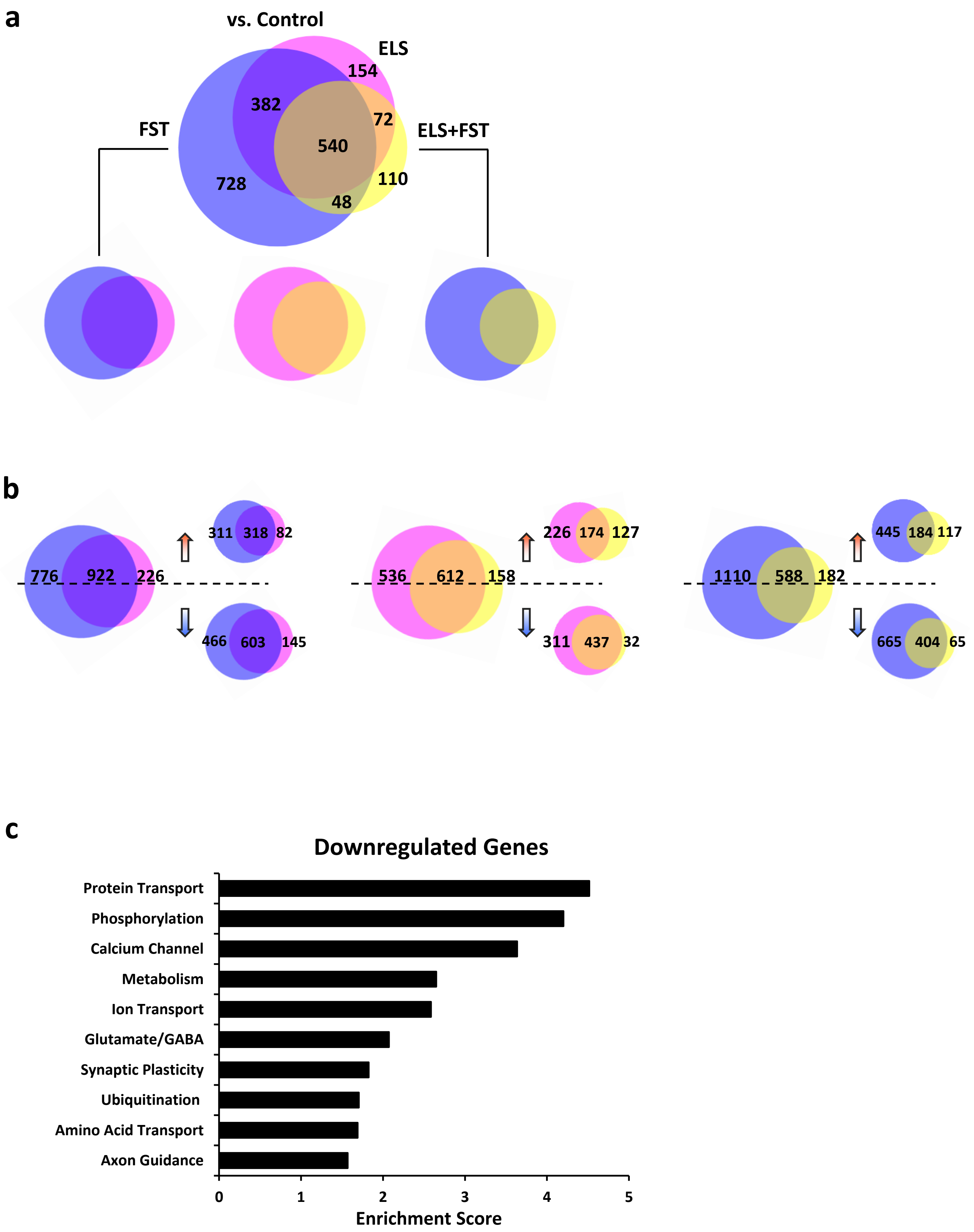

Supplementary Fig. 5

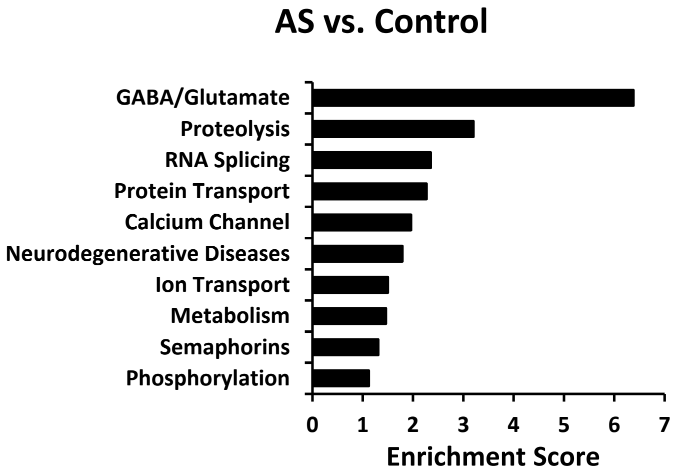

Supplementary Fig. 6

Genes	ELS+FST vs. FST	
	Fold Change	P-value
Scg2	0.62	0.015
Per1	0.66	0.001
Gadd45b	0.74	0.002
Sparcl1	0.78	0.005
Mal	0.87	0.041
Npy	0.88	0.024
Cst3	0.89	0.015
Dusp5	0.91	0.020
Dusp1	0.91	0.013
Trib1	0.94	0.015
Tiparp	0.95	0.015
Cldn11	0.97	0.025
Gadd45g	0.97	0.007
Egr1 *	0.98	2.9E-12
Htra1	1.04	0.015
Junb	1.04	1.4E-08
Rsrp1	1.06	3.8E-12
Nfkbia	1.08	0.015
Penk	1.08	0.007
Arl4d	1.12	1.2E-06
Dio2	1.17	0.043
Sik1	1.19	1.3E-05
Npas4	1.29	0.010
Egr4 *	1.29	1.0E-15
Arc *	1.29	7.5E-19
Sdc4	1.30	0.048
Ppp1r3g	1.37	0.019
Fosb *	1.39	4.3E-07
Nr4a1	1.40	7.4E-14
Plekhf1	2.14	0.008
Fos *	2.27	9.3E-30
Egr2 *	2.89	2.0E-14
Pttg1	-0.95	0.041
Ndufs5	-0.88	0.041

☐ **Genes common with FST vs. Control**

Table 1
